# Astrocytes at the interface of peri-ictal breathing dysfunction and SUDEP

**DOI:** 10.3389/fnetp.2026.1955271

**Published:** 2026-10-01

**Authors:** Isato Fukushi, Isamu Ozaki, Mieczyslaw Pokorski, Yasumasa Okada

**Affiliations:** 1 Graduate School of Health Sciences, Aomori University of Health and Welfare, Aomori, Japan; 2 Clinical Research Center, Murayama Medical Center, Musashimurayama, Japan; 3 Department of Rehabilitation Sciences, Hirosaki University of Health and Welfare, Hirosaki, Japan; 4 Department of Advanced Technology in Medicine, Institute of Biomedical Engineering, Institute of Science Tokyo, Tokyo, Japan; 5 Institute of Health Sciences, University of Opole, Opole, Poland

**Keywords:** astrocytes, epilepsy, glial cell, hypoxia, microglia, respiratory control, SUDEP, network physiology

## Abstract

Sudden unexpected death in epilepsy (SUDEP) is the leading cause of epilepsy-related mortality and is typically characterized by a terminal cascade of respiratory failure, cardiac arrhythmia, and cerebral suppression. Although epilepsy has long been regarded as a neuron-proprietary disorder, accumulating evidence indicates that glial cells, particularly astrocytes, play pivotal roles in both epileptogenesis and the progression to SUDEP. Astrocytes maintain neuronal homeostasis by regulating extracellular potassium through the inwardly rectifying potassium channel Kir4.1 and by clearing extracellular glutamate. Impairment of these homeostatic functions, together with reactive astrogliosis and the release of pro-inflammatory cytokines, including interleukin-1β and tumor necrosis factor-α, promotes neuronal hypersynchrony and lowers the seizure threshold. Severe hypoxia, a critical consequence in respiratory disturbance, sequentially activates microglia and astrocytes, thereby facilitating seizure generation and further depression of respiratory output. Furthermore, seizure propagation or spreading depolarization into brainstem respiratory centers, including the pre-Bötzinger complex and the nucleus tractus solitarius, may induce fatal central apnea or upper airway obstruction. Neuropathological evidence of neuronal and astrocytic loss in the ventrolateral medulla further suggests that chronic brainstem dysfunction increases susceptibility to respiratory failure. Experimental studies demonstrate that pharmacological inhibition of astrocytic activation delays hypoxia-induced seizures and respiratory arrest, highlighting astrocytes as a potential therapeutic target. A deeper understanding of neuron-glia interactions within the active neural milieu will facilitate the development of novel preventive strategies for SUDEP.

## 1 Introduction

Sudden unexpected death in epilepsy (SUDEP) is the most devastating sequela of epilepsy. SUDEP is defined as “death in a patient with epilepsy that is not due to trauma, drowning, status epilepticus, or other known causes but for which there is often evidence of an associated seizure” (Devinsky et al., 2018). The causes of SUDEP often remain unclear even upon autopsy (Sillanpää and Shinnar, 2010; Michalak et al., 2017). The pathogenetic mechanisms underlying SUDEP are not fully understood. However, converging evidence indicates that peri-ictal cardio-respiratory dysfunction and widespread cerebral suppression contribute to the fatal cascade (Ryvlin et al., 2013). Respiratory dysfunction is increasingly recognized as the primary determinant, as severe hypoxia and hypercapnic acidosis can suppress brainstem respiratory centers, leading to respiratory arrest.

Recent progress in glial physiology has revealed that astrocytes play an active role in information processing in various brain functions (Halassa and Haydon, 2010; Parpura et al., 2012), including ictogenesis (Vezzani et al., 2022; Purnell et al., 2023). The finding that glutamate release from astrocytes promotes local synchronous activity in hippocampal neurons via extrasynaptic NMDA receptors, implicating a role of astrocytes in generating epileptic discharges (Fellin et al., 2004). A study on an *in vitro* model of focal seizures has shown that recurrent excitatory loops between neurons and astrocytes shape seizure initiation and sustenance, as astrocytic Ca^2+^ elevation correlates with ictal, but not interictal discharges (Gómez-Gonzalo et al., 2010). These observations indicate that astrocytes may play an active, rather than merely supportive, role in epileptogenesis and peri-ictal respiratory dysfunction.

In this review, we summarize current evidence regarding the contribution of astrocytic dysfunction to epilepsy and examine the potential involvement of astrocytes in peri-ictal breathing abnormalities and SUDEP. We distinguish experimentally demonstrated mechanisms from novel proposed mechanistic pathways and highlight areas in which the available evidence remains limited or controversial. Particular attention is given to astrocyte–neuron and astrocyte–microglia interactions and their potential relevance to respiratory control. A better understanding of these interactions may provide a foundation for identifying novel strategies to stabilize respiratory network function and, ultimately, for developing targeted approaches to prevent or mitigate SUDEP.

## 2 Involvement of astrocytes in epileptogenesis

Epilepsy is a chronic neurological disorder characterized by a persistent predisposition to generate epileptic seizures and the associated neurobiological, cognitive, psychological, and social consequences (Fisher et al., 2005; 2017a; b). An epileptic seizure is defined as a transient occurrence of signs and symptoms resulting from abnormal or excessive neuronal activity in the brain. Traditionally, epilepsy was regarded as a disorder arising from overly neuronal excitability and synchronization within neuronal networks (Ransom and Blumenfeld, 2007). However, recent evidence has shown that glial cells, particularly astrocytes, actively contribute to epileptogenesis. A close functional interaction between neurons and glial cells has fundamentally changed understanding of epilepsy (Araque et al., 1999; Kurosinski and Götz, 2002; Magistretti, 2006; Barres, 2008; Allen and Barres, 2009; Xu et al., 2016). Epileptogenesis is now recognized as involving not only neuronal dysfunction but also disruption of bidirectional signaling between neurons and glia (Pollen and Trachtenberg, 1970; Glötzner, 1973; Bordey and Sontheimer, 1998; O’Connor et al., 1998; Heinemann et al., 2000; Perez Velazquez and Carlen, 2000; Steinhäuser and Seifert, 2002; D’Ambrosio, 2004; Tian et al., 2005; Binder and Steinhäuser, 2006; Li et al., 2007; Jabs et al., 2008; Wetherington et al., 2008; Lanerolle et al., 2010; Seifert et al., 2010; Aronica et al., 2012; Clasadonte et al., 2013; Devinsky et al., 2013; Vezzani et al., 2013; Verhoog et al., 2020; Grigorovsky et al., 2021; Heuser et al., 2021; Lentini et al., 2021; Sano et al., 2021; Wang et al., 2021). The recent concept of the active milieu emphasizes that seizures arise from an integrated cellular network—encompassing neurons, glia, vasculature, and extracellular matrix—rather than neuronal dysfunction alone (Semyanov and Verkhratsky, 2021).

Astrocytes regulate neuronal excitability by controlling extracellular [K^+^]_o_ and glutamate concentrations (Steinhäuser et al., 2012; Çarçak et al., 2023). Because the extracellular space is limited, neuronal activity rapidly alters [K^+^]_o_. During neuronal hyperactivity [K^+^]_o_ can rapidly rise to as high as 10–12 mM, the level observed during epileptiform activity. During neuronal activity, astrocytes restore [K^+^]_o_ homeostasis via K^+^ uptake and spatial K^+^ buffering (Kofuji and Newman, 2004). Spatial K^+^ buffering is driven by the difference between the membrane potential of the glial syncytium and the local K^+^ equilibrium potential. K^+^ enters astrocytes at sites where [K^+^]_o_ is elevated, and the astrocytic resting potential becomes more negative than the K^+^ equilibrium potential, whereas K^+^ exits at sites distant from the [K^+^]_o_ increase. This process transfers K^+^ through the syncytium from regions of high to low [K^+^]_o_. In astrocytes, the inwardly rectifying K^+^ channel Kir4.1, which is regulated by intracellular ATP, enables K^+^ influx at negative membrane potentials (Butt and Kalsi, 2006; Olsen and Sontheimer, 2008; Hibino et al., 2010). Animal studies have shown that Kir4.1 is essential for astrocytic K^+^ buffering (Djukic et al., 2007). When this buffering is impaired by Kir4.1 dysfunction or gap-junction uncoupling [K^+^]_o_ remains elevated, depolarizing the neuronal resting membrane potential and lowering the seizure threshold. Several studies have reported dysfunction or reduced expression of astrocytic Kir4.1 channels in humans and animal models of epilepsy (Kinboshi et al., 2020; Ohno et al., 2021). Conversely, increased Kir4.1 expression has been reported in mouse models of Dravet syndrome, which is a developmental epilepsy syndrome associated with drug-resistant seizures (Miljanovic et al., 2021), suggesting that astrocytic responses depend on disease context. Astrocytic electrogenic glutamate uptake is enhanced at hyperpolarized membrane potentials (Grewer and Rauen, 2005). Under physiological conditions, glutamate released into the synaptic cleft is efficiently taken up by astrocytes, converted to glutamine by glutamine synthetase, and recycled back to neurons, via a process known as the glutamate-glutamine cycle (Eid et al., 2016; Rose et al., 2018). Impaired glutamate reuptake allows excess glutamate to overstimulate neurons and trigger seizures (Eid et al., 2005; 2016; Seifert and Steinhäuser, 2013). Astrocyte-specific deletion of *Kcnj10*, the gene encoding Kir4.1, disrupts K^+^ and glutamate uptake and leads to increased seizure activity and premature death (Djukic et al., 2007).

Gliotransmitters released from astrocytes—glutamate, D-serine, and ATP—effectively modulate synaptic transmission to neurons. Normally, astrocytes remain quiescent in the brain. However, neurological insults, such as traumatic brain injury, stroke, status epilepticus, and viral infections, induce reactive astrogliosis, a hallmark of the epileptic focus (Devinsky et al., 2013; Binder and Steinhäuser, 2021). Reactive astrocytes release pro-inflammatory cytokines, including interleukin-1β and tumor necrosis factor-α, which reinforce epileptogenic inflammatory signaling (Wetherington et al., 2008; Aronica et al., 2012; Verhoog et al., 2020), playing a key role in seizure initiation (Sano et al., 2021; Vezzani et al., 2022; Purnell et al., 2023). Recurrent excitatory loops between astrocytes and neurons facilitate seizure initiation and sustenance via astrocytic Ca^2+^ elevation, which correlates with the initiation and sustenance of ictal, but not interictal discharges. (Gómez-Gonzalo et al., 2010). A recent study demonstrated that optogenetic activation of channelrhodopsin-2-expressing astrocytes exerts an anti-seizure effect on kainic acid-induced neocortical seizures (Zhao et al., 2022). This anti-seizure effect is driven by the stimulation of astrocytic Na^+^-K^+^-ATPase resulting from Na^+^ influx, independent of Ca^2+^ signaling. Collectively, these findings indicate that astrocytes exhibit marked functional heterogeneity and can either promote or suppress seizure activity depending on the pathological context and underlying molecular mechanisms (Okada et al., 2026).

## 3 Astrocytic and respiratory mechanisms underlying hypoxia-induced ictogenesis and SUDEP

### 3.1 Severe hypoxia links seizures to respiratory failure

Respiratory compromise is regarded as the final pathway leading to SUDEP. Among various factors implicated in this process, hypoxia occupies a central place, impairing respiratory function and promoting seizure generation through glial activation. Emerging evidence shows astrocytes as critical links to hypoxia, epileptogenesis, and respiratory failure. Hypoxia plays a central role by impairing respiratory function and promoting seizures via glial activation, with astrocytes serving as critical links among hypoxia, epileptogenesis, and respiratory failure. This link finally leads to SUDEP.

Mild hypoxia increases ventilation through carotid body activation (Izumizaki et al., 2004; Kumar and Prabhakar, 2012). Brainstem astrocytes contribute to hypoxic ventilatory augmentation by directly sensing hypoxia and enhancing respiratory output via gliotransmission (Tadmouri et al., 2014; Angelova et al., 2015; Fukushi et al., 2016; Marina et al., 2016; 2018; Pokorski et al., 2016; Gourine and Funk, 2017; Rajani et al., 2018; Sheikhbahaei et al., 2018; Uchiyama et al., 2020). Conversely, severe hypoxia inhibits the brainstem respiratory circuit following initial augmentation (Blum et al., 2000; So, 2008), causing diminished ventilation, exacerbated hypoxia, and global brain suppression (Fukushi et al., 2016; 2020). It deepens ventilatory depression, creating a vicious cycle that culminates in respiratory arrest and death (Neubauer et al., 1990; Kikuchi et al., 1994; Hayashi and Fukuda, 2000; Fukushi et al., 2016; 2020).

We previously reported that pretreatment with arundic acid, an inhibitor of astrocytic activation, delays the onset of severe hypoxia-induced seizures and respiratory arrest in mice (Fukushi et al., 2020) (Figure 1). This finding provides experimental evidence for a potential contribution of astrocytic activation to hypoxia-induced seizures and respiratory arrest. We hypothesize that this effect may involve excessive activation of forebrain astrocytes during severe hypoxia (Leichsenring et al., 2013; Fukushi et al., 2020), which could promote neuronal hyperexcitability and hypersynchrony within neuronal networks (Avoli et al., 2002; Stafstrom, 2006; Crunelli et al., 2015), thereby facilitating seizure generation and, potentially, seizure-induced respiratory arrest and death. However, this proposed pathway remains mechanistic and has not been established as a direct causal sequence *in vivo*. Conversely, neuropathological analysis in SUDEP cases revealed a marked reduction in vimentin-positive glia within the ventrolateral medulla and lateral medullary subpial layer, together with decreased connexin-43 expression in the median raphe (Patodia et al., 2019). These findings suggest an association between astrocytic abnormalities and increased susceptibility to fatal respiratory dysfunction, although a direct causal relationship remains to be established (Aiba and Noebels, 2015; Loonen et al., 2019; Noebels, 2019). Collectively, these observations raise the possibility that both excessive and insufficient astrocytic activity may adversely affect respiratory control under pathological conditions (Okada et al., 2026). Further experimental studies are therefore needed to determine whether and how astrocyte-dependent pathways causally contribute to SUDEP pathogenesis.

**FIGURE 1:**
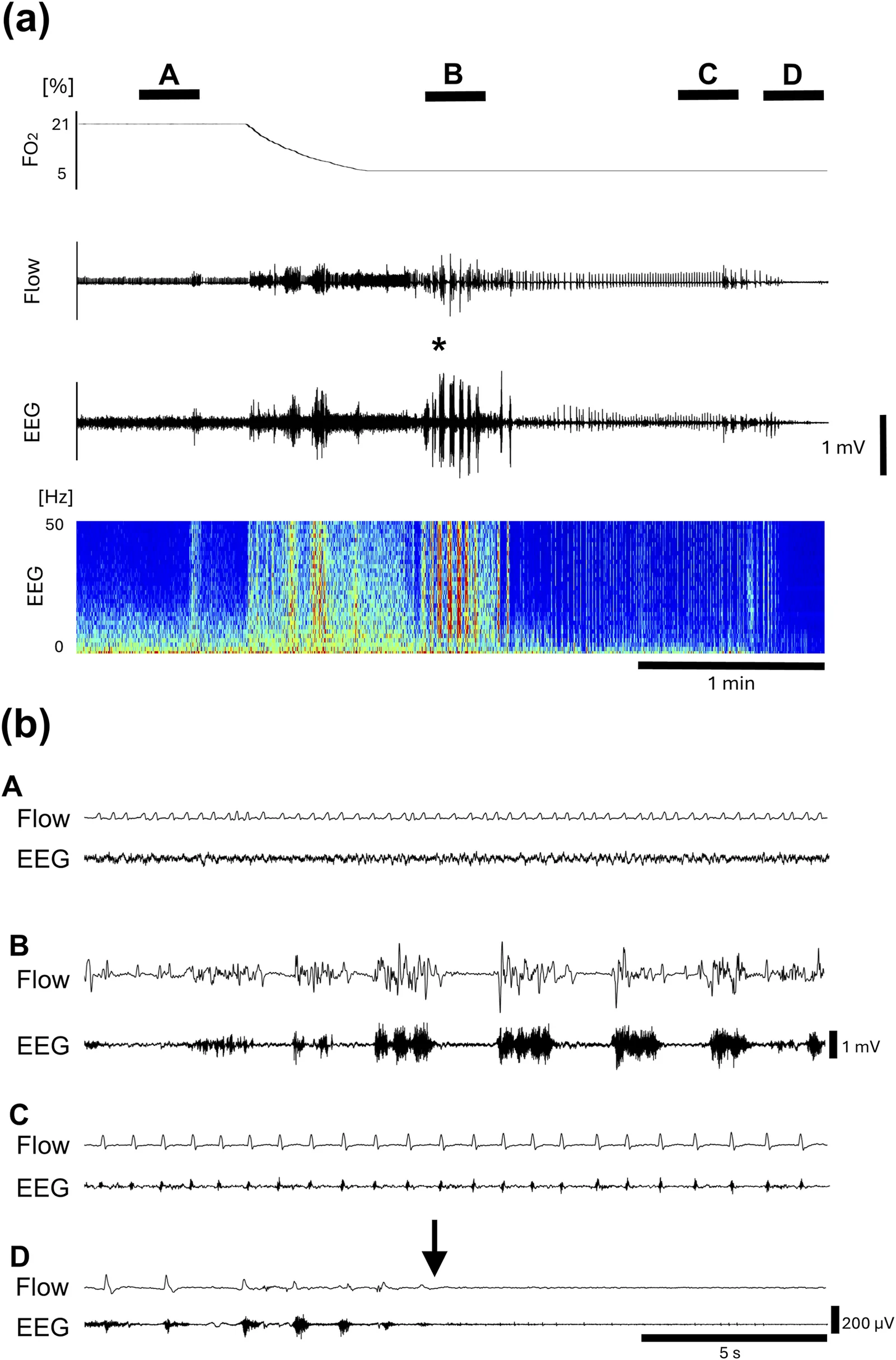
Representative recordings in hypoxia loading experiments. **(a)** From top to bottom: oxygen concentration in a chamber, respiratory flow (whole body plethysmogram, inspiration upward), EEG raw signal, and EEG spectrogram. Bars A, B, C, and D in **(a)** correspond with the expanded periods A, B, C, and D in **(b)**, respectively. Seizure activity was indicated on the EEG trace with an asterisk (*). **(b)** Expanded traces. **(A)** Room air condition. **(B)** Hypoxic condition around the time of seizure appearance. **(C)** Hypoxic condition around the time of ventilatory depression. **(D)** Hypoxic condition around the time of respiratory arrest. We observed gasping just before the occurrence of respiratory arrest and EEG flatlining. The time of respiratory arrest was indicated with an arrow. Adopted from Fukushi et al. (2020) with permission from John Wiley & Sons.

### 3.2 Sequential activation of microglia and astrocytes

Microglial and astrocytic activation is a well-described manifestation of epilepsy (Beach et al., 1995; Boer et al., 2006; Devinsky et al., 2013), where astrocytic dysfunction drives neuronal hyperexcitability and seizures (Coulter and Steinhäuser, 2015; Fukushi et al., 2020). Microglial activation precedes reactive astrogliosis in various neurological disorders (Sofroniew and Vinters, 2010; Shinozaki et al., 2014).

Severe hypoxia activates microglia (Fukushi et al., 2023). Upon activation, microglia release ATP, which binds to P2Y_1_ receptors on astrocytes, stimulating neighboring astrocytes in a paracrine manner (Pascual et al., 2012). Activated microglia lower the seizure threshold by promoting pro-inflammatory cytokines and astrocytic glutamate release (Volterra and Meldolesi, 2005; Vezzani et al., 2011; Galic et al., 2012; Vezzani et al., 2013; Benson et al., 2015). Thus, hypoxia-enhanced microglial activation in brain regions critical for epileptogenesis drives seizure onset and the subsequent astrocytic activation, precipitating postictal ventilatory suppression (Fukushi et al., 2023). The sequential activation of microglia and astrocytes is a key in epileptogenesis and seizure treatment schemes in epileptic models (Sano et al., 2021).

### 3.3 Brainstem respiratory dysfunction

Brainstem structures—pre-Bötzinger complex, parafacial respiratory group/retrotrapezoid nucleus, nucleus tractus solitarius (NTS)—are essential for maintaining adequate respiratory regulation (Smith et al., 1991; Onimaru and Homma, 2003). Neuropathological studies have consistently demonstrated a volume loss of the ventrolateral medulla in SUDEP victims, accompanied by reductions in neurons and astrocytes. The volume loss of the rostral medulla deepens as epilepsy progresses, which implicates it in postictal respiratory compromise in SUDEP (Mueller et al., 2014; Patodia et al., 2019; 2021).

Seizures originate in the cortex, with lethality involving disruption of autonomic and respiratory function at the brainstem level (Thijs et al., 2021; Teran et al., 2022). Several mechanisms have been proposed for the propagation of cortical seizures to the brainstem. Brainstem respiratory centers maintain reciprocal connections with multiple cortical and subcortical structures, providing an anatomical substrate for respiratory modulation (Smith et al., 1991; Harper et al., 1998; McKay et al., 2003; Bondarenko et al., 2014; Ashhad et al., 2022; Feinstein et al., 2022). Interictal cardiorespiratory problems are common in epilepsy (Barot and Nei, 2019). Seizure propagation into brainstem respiratory areas suppresses their function, triggering central apneas (Bateman et al., 2008). Specifically, the hyperexcitability of GABAergic neurons in the NTS reduces the threshold for spreading depolarization, a neuronal event facilitating acute respiratory and cardiac shutdowns (Su et al., 2008; Aiba and Noebels, 2015; Derera et al., 2017).

### 3.4 Depolarization spread and SUDEP

The spread of depolarization propagates waves of near-complete neuronal excitation accompanied by changes in ionic and neurotransmitter homeostasis (Pietrobon and Moskowitz, 2014; Cozzolino et al., 2018). Increases in extracellular [K^+^]_o_ and glutamate initiate a self-propagating wave of depolarization and cytotoxic edema (Hinzman et al., 2016; Hubel et al., 2017). Depolarization propagation through contiguous tissue is strongly influenced by ongoing neural activity and metabolic status (Aiba and Noebels, 2015). It is shortly followed by neural inactivation, which suppresses EEG activity, as is frequently observed after generalized tonic-clonic seizures (GTCS) (Surges et al., 2011). In the cortex, spreading depression harms cardiorespiratory control. GTCS are the common type of seizures associated with SUDEP, underlain by dysregulation of the ascending reticular activating system and descending reticulospinal projections (Sedigh-Sarvestani et al., 2015; Ryvlin et al., 2019). Once initiated, the spread of depolarization affects neuronal activity and contributes to seizure-induced mortality. However, the association between postictal generalized EEG suppression (PGES), which has been proposed to reflect widespread neuronal dysfunction following seizures, and SUDEP remains controversial. Accordingly, whether PGES can serve as a reliable biomarker of SUDEP risk remains a matter of debate (Lhatoo et al., 2010; Surges et al., 2011; Lamberts et al., 2013; Moseley and DeGiorgio, 2015). Surges et al. (2011) demonstrated that the occurrence and duration of PGES varied across seizures within individual patients, suggesting that PGES may represent a seizure-specific, transient phenomenon rather than a stable, trait-like marker of SUDEP risk. Consistent with this view, Ryvlin et al. (2013) suggested that the pathophysiological processes underlying SUDEP may extend beyond PGES itself and involve a rapid failure of cardiorespiratory and autonomic control, manifested by early postictal respiratory dysfunction and/or terminal ventricular arrhythmias. Collectively, these findings underscore the limitations of PGES as a standalone biomarker for predicting SUDEP risk and highlight the need to consider PGES in the broader context of postictal cardiorespiratory and autonomic dysfunction.

Epilepsy-associated genetic mutations, which promote cortical seizure activity, may also be expressed in the brainstem, where they increase the risk of SUDEP in seizure-dependent and independent manners (Kuo et al., 2019). Animal studies showed that cortical seizure activity causes brainstem dysfunction and SUDEP, although not necessarily leading to premature death (Cheah et al., 2012; Aiba and Noebels, 2021). The reason for these divergent results is unclear. Epilepsy-associated genes are expressed by neurons and astrocytes in the brainstem respiratory system, which makes it vulnerable to failure.

### 3.5 Respiratory mechanisms leading to SUDEP

Seizure-related autonomic dysfunction that includes respiratory control centers is consistently linked to hypoventilatory disorders. Hypoxia that follows respiratory dysfunction worsens neurological injury and respiratory depression and raises SUDEP risk (Devinsky, 2004; Villiere et al., 2017; Nobis et al., 2019; Mulkey and Milla, 2022). Temporal lobe epilepsy (TLE) involves limbic structures in apnea and oxygen desaturation (Bateman et al., 2008; Lacuey et al., 2018). In TLE mouse models, Kv4 potassium current reduction causes GABAergic neuronal hyperexcitability in the NTS, which is vital for cardiorespiratory integration (Bailey et al., 2007; Su et al., 2008; Lee et al., 2014; Strube et al., 2015; Villa and Combi, 2016; Derera et al., 2017). This hyperexcitability may trigger respiratory and cardiac dysfunction, contributing to SUDEP. The amygdala sends extensive inhibitory projections to brainstem respiratory centers (Feldman et al., 2003; Nardi et al., 2009; Feinstein et al., 2022). Stimulatory seizure spread to the amygdala may trigger apnea (Dlouhy et al., 2015; Lacuey et al., 2017; Gu et al., 2024). However, seizures originating in the amygdala do not necessarily result in apnea (Park et al., 2020). SUDEP often begins with terminal GTCS, followed first by tachypnea and then by apnea that culminates in respiratory arrest (Smart et al., 1998). In this pattern, failed chemosensory response to hypoxia leads to apnea despite preceding hyperventilation (Xie et al., 1994; 1997; Kim et al., 2018). Prolonged hypoxia can cause cardiac arrest within minutes (Li et al., 2015).

Obstructive apnea and bradycardia may act synergistically to trigger SUDEP. Obstructive apnea is also a central driver in the SUDEP cascade. Laryngospasm, resulting from seizure-associated hyperactivity of the recurrent laryngeal nerve, causes airway obstruction and rapidly worsening hypoxia. The intense autonomic response during attempted breathing against a closed airway drives cardiopulmonary collapse (Stewart et al., 2017; 2020). Seizures spreading from the subiculum to the hypothalamic paraventricular nucleus and medulla activate respiratory and autonomic centers, contributing to laryngospasm and apnea (Geerling et al., 2010; Peng et al., 2017).

Hypoventilation during seizures is a significant cause of SUDEP. It impairs CO_2_ clearance and contributes to hypoxemia and hypercapnia, exacerbating seizure effects. In animal models, hypoventilation commonly occurs during ictal and postictal phases, due to the malfunction of respiratory centers or altered baseline breathing (Azar et al., 2008; Bateman et al., 2008; Seyal et al., 2013; Moore et al., 2014). Hypoventilation often co-occurs with apnea, impairing adenosinergic and serotonergic signaling, spreading depolarization, and autonomic dysregulation (Paydarfar et al., 1991; 1992; During and Spencer, 1992; Faingold et al., 2010; Lutas and Yellen, 2013; Feng and Faingold, 2017).

Building on these collective insights, we propose an integrative model outlining potential glial and respiratory mechanisms underlying SUDEP pathogenesis. SUDEP is frequently initiated by terminal GTCS. Upon GTCS onset, seizure activity propagates to the brainstem, sparking astrocytic dysfunction. Compromised astrocytic potassium buffering impedes the clearance of elevated [K^+^]_o_ during neuronal activity, culminating in sustained high [K^+^]_o_ and a lowered seizure threshold. The astrocytic impairment attenuates CO_2_ sensitivity and the hypercapnic ventilatory response while suppressing the release of gliotransmitters, such as ATP, which collectively destabilize respiratory regulation. The propagation of seizure activity within the brainstem disrupts central autonomic networks, triggering central and obstructive apneas and hypoventilation, which lead to acute ventilatory failure. Intertwined astrocytic and respiratory disturbances contribute to hypoxia and hypercapnia, precipitating cardiorespiratory collapse and SUDEP (Figure 2). This evidence suggests that astrocytic cell malfunction and breathing deficit trap the body in a harmful loop, pushing epilepsy further toward sudden death.

**FIGURE 2:**
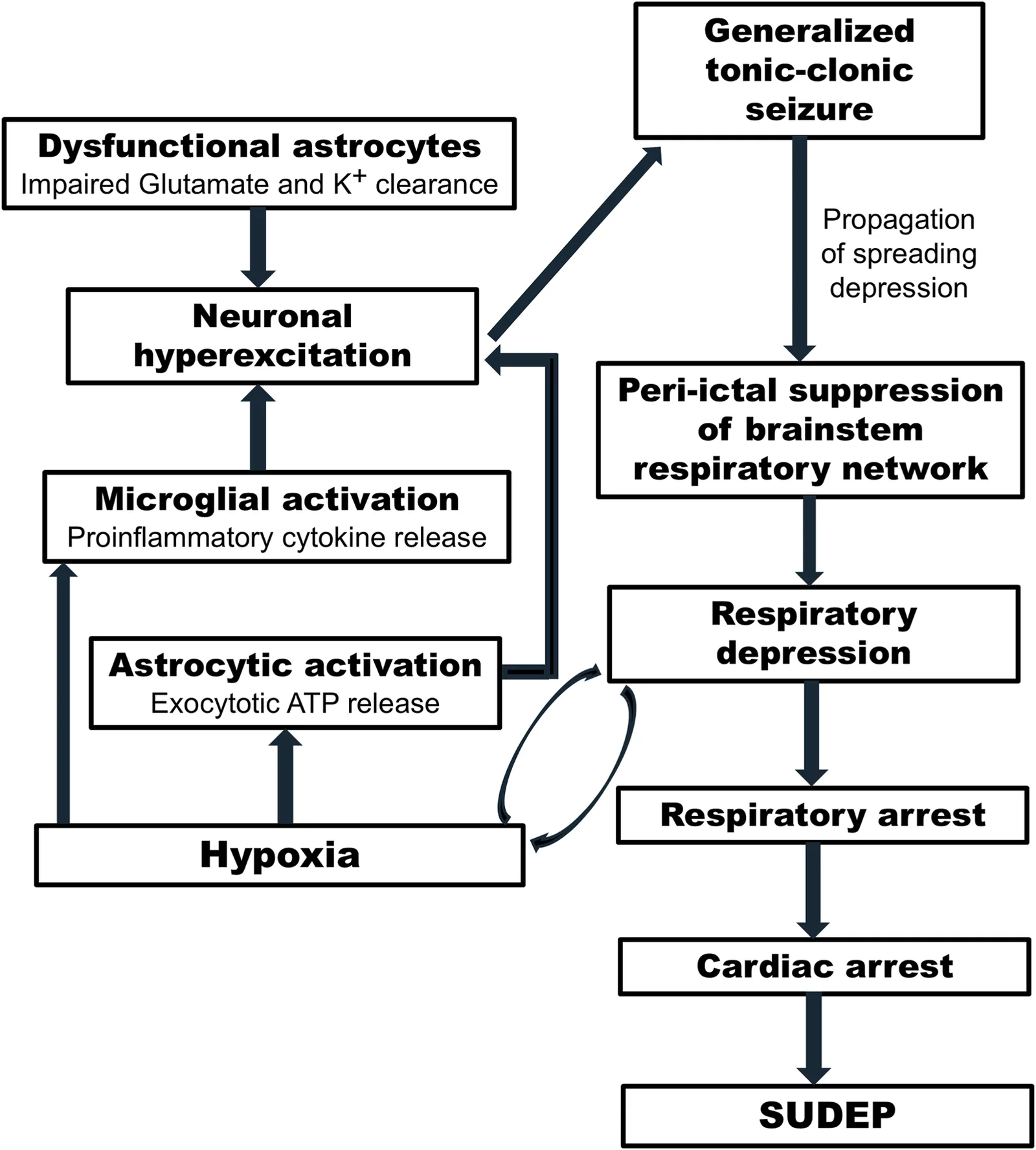
Schematic diagram of glial and respiratory mechanisms leading to SUDEP. Hypoxia serves as a key initiator, triggering microglial activation and the subsequent release of pro-inflammatory cytokines. This microglial response, combined with hypoxia-induced astrocytic activation and exocytotic ATP release, drives neuronal hyperexcitation. Concurrently, astrocytic dysfunction—characterized by impaired glutamate and K^+^ clearance—destabilizes the extracellular environment, lowering the threshold for generalized tonic-clonic seizures (GTCS). Following GTCS, the spread of depression propagates toward the brainstem, causing peri-ictal suppression of the brainstem respiratory network. The resulting respiratory depression is further exacerbated by worsened hypoxia, establishing a self-reinforcing pathological loop. The collapse of brainstem autonomic and respiratory control centers ultimately leads to respiratory arrest and subsequent cardiac arrest, culminating in SUDEP.

## 4 Future perspectives

Growing evidence shows that astrocytes are active players in epileptogenesis and seizure progression to SUDEP. Nevertheless, the precise cellular and molecular mechanisms by which astrocytes initiate, amplify, or suppress seizure activity have yet to be fully elucidated, as do the pathways governing cortical seizure propagation to brainstem respiratory centers and cardiorespiratory collapse.

An unresolved issue is whether astrocytic epileptic alterations are the driving pathogenic force or secondary responses to recurrent seizures. Additionally, it remains unclear if epilepsy-associated genes expressed in neurons or astrocytes within brainstem respiratory networks contribute directly to respiratory susceptibility to SUDEP. How dysregulated astrocytic potassium buffering, glutamate clearance, and gliotransmission influence seizure propagation into the brainstem requires further clarification. Given the functional heterogeneity of astrocytes, which can either facilitate or suppress seizures through distinct pathways, identifying the mechanisms governing these opposing phenotypes is critical for discovering novel therapeutic targets.

Future therapeutic strategies for epilepsy and SUDEP are likely to extend beyond neurons, targeting glial cells. Restoring astrocytic homeostasis, including potassium buffering, glutamate clearance, gliotransmission, and the regulation of neuroinflammation, opens up novel avenues for preventing seizure progression and reducing the risk of SUDEP. Additionally, elucidation of bidirectional communication between astrocytes and microglia may help identify new therapeutic interventions. A more comprehensive understanding of neuron-glia interactions in the active neuronal milieu will advance understanding of epileptic pathophysiology and accelerate the development of targeted preventative and therapeutic measures.
